# Editorial for Special Issue “Research on Friction, Wear and Corrosion Properties of Materials”

**DOI:** 10.3390/ma19102044

**Published:** 2026-05-13

**Authors:** Yucheng Liu, Yunhai Ma

**Affiliations:** 1 Key Laboratory of Modern Agricultural Equipment and Technology, Ministry of Education, Jiangsu University, Zhenjiang 212013, China; 2 Key Laboratory of Bionic Engineering, Ministry of Education, Jilin University, Changchun 130022, China

## 1. Introduction and Scope

In the field of materials engineering, friction, wear, and corrosion are key factors that constrain the service performance and lifespan of materials, and have long been extensively studied by both academia and industry [1,2,3,4]. Friction not only manifests as a mechanical response that hinders the relative motion of contacting surfaces but also serves as the primary source of system energy dissipation. Wear is a gradual loss of surface material induced by mechanical effects, while corrosion involves harmful chemical reactions between materials and environmental media. The synergistic effects of these three factors often significantly accelerate the failure and destruction of components. For instance, in marine engineering equipment or human biological implants, the synergistic effects of mechanical wear and chemical/electrochemical corrosion often lead to material loss far exceeding that caused by a single factor [5,6]. Therefore, researchers need to deeply reveal the intrinsic mechanisms of material friction, wear, and corrosion, and then develop advanced materials with high wear and corrosion resistance and low friction coefficients. This has significant engineering value and application prospects for reducing energy loss, ensuring the safe operation of equipment, and enhancing equipment durability. Currently, scholars both domestically and internationally have conducted extensive research on the tribological properties and corrosion behavior of materials [7,8,9]. Through multi-scale characterization and exploration of microscopic mechanisms, a series of damage evolution laws have been elucidated, and various surface modification and alloy design strategies have been proposed to optimize material properties [10,11,12]. However, the complex failure mechanisms induced by the coupling of multiple factors under extreme conditions have not been fully explored, and existing protective measures are difficult to meet increasingly stringent engineering demands. There is an urgent need to further explore the essential laws of material loss and degradation, providing solid theoretical and technical support for the development of a new generation of high-performance long-life materials.

The special issue “Research on Friction, Wear and Corrosion Properties of Materials” compiles the latest research findings on the friction, wear, and corrosion of materials, particularly focusing on alloy friction and wear, as well as coating corrosion resistance. The topics covered in the Special Issue mainly include friction materials, heterogeneous structural materials, resistance and wear reduction, material bionics, friction properties, wear properties, corrosion properties, flame spraying, and related applications.

## 2. Overview of Published Articles

The Special Issue includes seven research papers and one review paper by authors from China, Poland, and Brazil.

For the first research paper, Che et al. [13] prepared *β*-tricalcium phosphate powders with different strontium (Sr)/silver (Ag) doping levels using the sol-gel method. After optimizing the ratio of pore-forming agent and binder, porous ceramic samples were obtained by sintering in a muffle furnace at 1000 °C for 5 h. Through in vitro degradation experiments in simulated body fluid and a series of characterizations, it was found that Sr/Ag doping significantly regulates the crystallinity and structural parameters of the material. Although the overall compressive strength decreased after degradation, the mechanical properties of the doped group were superior to those of the undoped group, and a apatite-like materials was successfully induced on the surface of all samples. Sr/Ag co-doped *β*-tricalcium phosphate combines excellent osteogenic activity with suitable mechanical strength, making it a promising bone repair biomaterial for clinical applications.

For the second research paper, Ma et al. [14] were inspired by the intricate multi-scale porous network within wood cells and developed a bionic sound-absorbing structural material with a hierarchical porous structure. They demonstrated that the bionic coupled structure significantly enhances noise friction and sound absorption effects. These perforated resonators produce complementary frequency responses and porous propagation effects, ultimately achieving broadband noise attenuation. Samples with controllable parameters such as pore size, porosity, number of layers, and layer sequence were fabricated using a 3D printing device. Acoustic impedance tube characteristic tests confirmed that this structure can achieve broadband and efficient sound attenuation by customizing impedance matching and enhancing viscous loss. Under optimized parameters, the sound absorption coefficient approaches 1, representing an average sound absorption rate improvement of 25–35% and the operational bandwidth is broadened by approximately 95%, compared to the single-hole baseline. This research systematically reveals the correlation criteria between porous biological morphology and acoustic performance, providing an important reference for the application of various materials and equipment in noise control and dissipation engineering.

Addressing the challenge of the mud pulser rotor failing due to long-term scouring action from solid particles and corrosive slurry, Contribution 1 conducted deep cryogenic treatment (DCT) at −196 °C for 2.5 h on three alloy samples with different cobalt contents: WC-5Co, WC-8Co, and WC-10Co. They then systematically tested and characterized these samples. DCT significantly promoted the precipitation of *η* phase and the enhancement of *ε*-Co diffraction peaks. Meanwhile, the wear rates of WC-5Co, WC-8Co, and WC-10Co were significantly reduced by 14.71%, 37.25%, and 41.01%, respectively. Additionally, it shifted the corrosion potential forward and decreased the corrosion current density. The improvement in wear resistance was primarily attributed to the strengthening of the *η* phase and the increase in residual compressive stress in WC, which inhibited the extrusion deformation and spalling of cobalt and WC. The enhancement in corrosion resistance stemmed from the combined optimization of electrode potential by cobalt phase transformation, *η* phase precipitation, and increased compressive stress in the alloy. This work provides important low-temperature modification insights for the surface strengthening of cemented carbides under extreme conditions.

Contribution 2 systematically investigated the tribological behavior of precipitation hardened and cryogenic treatment multiplex heat treated WE43 and WE54 magnesium-rare earth alloy in reciprocating linear sliding contact with AISI 316-L steel, Si_3_N_4_, WC tungsten carbide and ZrO_2_. The multiplex heat treatment significantly optimized the wear resistance of the alloys, with the best anti-friction and anti-wear effect achieved when paired with sample 316-L steel, resulting in a nearly 2-fold reduction in specific wear rate. Combined with microscopic analysis of wear morphology, the wear mechanism and product evolution were clarified. By improving the standard algorithms of ASTM G133 and D7755, precise wear quantitative measurement was achieved, establishing “cryogenic + precipitation hardening” as the optimal process for enhancing the durability of this series of alloys. Moreover, it provided a standardized and experimentally verified testing method for evaluating the tribological properties of magnesium-rare earth alloy.

Contribution 3 addressed the core issue of tool wear limiting the quality of sliced wafers in diamond wire saw processing of monocrystalline silicon wafers. The authors employed a single-factor experimental method to investigate the influence patterns and mechanisms of changes in wire speed, feed rate, and workpiece thickness on diamond wire wear. The study revealed that diamond wire wear experiences a steep increase in both the initial and final stages, corresponding to a significant deterioration in the quality of the processed wafers. However, during the stable wear phase, the wear rate stabilizes, allowing for the production of high-quality wafers. Under a constant processing area of the workpiece, the wear amount of the diamond wire decreases as the wire speed increases, but significantly increases with the increase in feed rate and workpiece thickness. Notably, when the processing wire speed and feed rate are increased proportionally, the wear amount of the diamond wire remains essentially constant.

Contribution 4 employed electrochemical impedance spectroscopy technology for underwater measurements to assess the resistance of the epoxy coating on the legs of an oil production platform in seawater environment. By comparing the results of field measurements offshore with the coating thickness, hardness, adhesion, and resistance values obtained from laboratory simulations, the reliability of the underwater painting process by divers was verified. The resistance value of the single-layer epoxy coating brushed onto the platform legs exceeded 10 kΩ·cm^2^, meeting the minimum resistance threshold required for synergistic corrosion protection between cathodic protection and coating. This conclusion was strongly supported by the potential monitoring data of the platform legs: the potential values of the platform legs (at depths of 0–15 m) significantly decreased from 310–320 mV to 220–240 mV relative to the zinc reference electrode after coating, achieving effective comprehensive cathodic protection for the platform legs. The underwater electrochemical impedance spectroscopy measurement method developed in this study can effectively evaluate the quality of any underwater metal structure coating.

Contribution 5 systematically evaluated the corrosion behavior of four aftermarket additive products on high-lead tin bronze alloys over a period of 28 days under 80 °C water immersion test conditions, focusing on the corrosion-related risks that may arise from the application of aftermarket additives in internal combustion engines. Among them, three aftermarket additives caused moderate to severe corrosion damage, and relying solely on surface appearance observation can easily underestimate the actual corrosion hazards. Accurate determination must be made through chemical analysis of the oil drainage and substrate surface. The corrosion does not originate from the degradation of base oil, but rather from direct interfacial interactions between sulfur- and chlorine-containing additives and alloy metal elements, with a significant correlation between dissolved metals and sulfur- and chlorine-containing additives adsorbed on the surface. It is worth noting that when chlorine-containing additives are mixed with SAE 10W-30 gasoline engine oil at a ratio of 25:1, their corrosion effects are significantly inhibited, confirming that correct mixing can effectively mitigate the corrosion tendency of individual additives.

In view of the vast potential of layered double hydroxides (LDHs) in marine corrosion protection, Contribution 6 systematically reviewed the latest research progress of their application in various functional coatings. Based on a brief description of the preparation methods and properties of LDHs, they focused on analyzing the mechanisms by which LDH-based composite coatings enhance corrosion resistance through physical barriers, self-healing, chloride trapping effects, and hydrophobic effects. They delved into the key factors affecting the corrosion resistance of composite coatings and pointed out the challenges and future prospects of LDH-modified composite coatings in the field of corrosion protection, providing an important theoretical basis for the development of a new generation of efficient protective materials suitable for various application scenarios.

## 3. Conclusions

This Special Issue provides a comprehensive overview of the latest innovative achievements in the friction, wear, and corrosion properties of materials, with contributions ranging from friction sound absorption, alloy friction, and wear to alloy corrosion prevention and coating corrosion prevention. These diverse research results highlight the importance of studying the friction, wear, and corrosion properties of materials and their mechanisms in engineering applications. More specifically, this Special Issue includes seven research papers and one review paper written by experts in their respective fields and peer-reviewed. These studies have solved many practical problems and provided reference and guidance for future research work in this field. With the efforts of experts and editors, the first volume has had a wide-ranging social impact. Moreover, in response to strong suggestions from researchers, we have started the essay solicitation work for the second volume. We warmly welcome everyone to publish high-quality and innovative achievements in this field in the second volume of this Special Issue. This Special Issue will become an important platform for researchers and engineers to exchange, cooperate, and promote the rapid development of this field and make outstanding contributions to the industrialization of scientific research achievements.
